# Action of GABAB receptor on local network oscillation in somatosensory cortex of oral part: focusing on NMDA receptor

**DOI:** 10.1186/s12576-024-00911-w

**Published:** 2024-03-12

**Authors:** Hiroyuki Kanayama, Takashi Tominaga, Yoko Tominaga, Nobuo Kato, Hiroshi Yoshimura

**Affiliations:** 1 Department of Molecular Oral Physiology, Institute of Biomedical Sciences, Tokushima University Graduate School, 3-18-15 Kuramoto, Tokushima, 770-8504 Japan; 2 Institute of Neuroscience, Tokushima Bunri University, Shido, Kagawa 769-2123 Japan; 3 Department of Oral and Maxillofacial Surgery, National Hospital Organization Osaka National Hospital, Osaka, 540-0006 Japan; 4 Department of Physiology, Kanazawa Medical University, Uchinada-Cho, Ishikawa, 920-0293 Japan

**Keywords:** Oral somatosensory cortex, GABA_B_ receptor, Network oscillation, Caffeine, NMDA receptor, Voltage-sensitive dye

## Abstract

The balance of activity between glutamatergic and GABAergic networks is particularly important for oscillatory neural activities in the brain. Here, we investigated the roles of GABA_B_ receptors in network oscillation in the oral somatosensory cortex (OSC), focusing on NMDA receptors. Neural oscillation at the frequency of 8–10 Hz was elicited in rat brain slices after caffeine application. Oscillations comprised a non-NMDA receptor-dependent initial phase and a later NMDA receptor-dependent oscillatory phase, with the oscillator located in the upper layer of the OSC. Baclofen was applied to investigate the actions of GABA_B_ receptors. The later NMDA receptor-dependent oscillatory phase completely disappeared, but the initial phase did not. These results suggest that GABA_B_ receptors mainly act on NMDA receptor, in which metabotropic actions of GABA_B_ receptors may contribute to the attenuation of NMDA receptor activities. A regulatory system for network oscillation involving GABA_B_ receptors may be present in the OSC.

## Introduction

The brain comprises a huge number of neurons, with the connections between individual neurons via synapses resulting in the formation of neural networks of various sizes. Information processing is based on interactions between neurons through feedback and feedforward signal communications [1–4]. One strategy for information processing is thought to involve encoding information with neural oscillations as rhythmic or repetitive patterns of synchronized fluctuations in membrane potential [5–7]. In neural network dynamics, the balance of activity between excitatory and inhibitory networks is particularly important for the generation of oscillations [8–10], and inhibitory networks play a key role in creating brain rhythms [7, 11, 12].

Actions of inhibitory network system are established by the neurotransmitter gamma-aminobutyric acid (GABA) and its two types of receptors, GABA_A_ and GABA_B_ receptors [13]. GABA_A_ receptors are linked to Cl^−^ channels and modulate membrane potential [14, 15]. GABA_B_ receptors are coupled with G-proteins and modulate neural activity by driving intracellular signaling pathways [16–19]. In the course of making rhythms, GABA_A_ receptor-mediated fast inhibition is crucial for the mechanisms underlying the synchronization of neuronal activity in cortical local networks [11, 19, 20]. However, the roles of GABA_B_ receptors, which provide slow synaptic modulation of network oscillations, have remained unclear.

Previous studies have revealed that GABA_B_ receptors are expressed at presynaptic terminals, the postsynaptic spine and extra-synaptic membranes [21–24]. GABA_B_ receptors are also expressed on interneurons, suggesting complex functions in multiple pathways [25, 26]. Presynaptic GABA_B_ receptors act to decrease transmitter release by attenuating voltage-gated Ca^2+^ channel (VGCC) activities in presynaptic terminals [27] and decreasing spontaneous transmitter release by inhibiting adenylyl cyclase (AC) [26]. Postsynaptic GABA_B_ receptors act to decrease membrane potential by activating G-protein-activated inwardly rectifying potassium channels (GIRKs), and by alleviating the tonic inhibition of TREK2 channels, a type of two-pore domain potassium channel [26, 28, 29]. Activation of GABA_B_ receptor also inhibits dendritic VGCCs, preventing dendritic Ca^2+^ spikes [30]. In addition, activation of GABA_B_ receptors has been demonstrated to affect *N*-methyl-d-aspartate (NMDA) receptors by driving the intracellular cyclic adenosine monophosphate (cAMP) pathway [30–33], resulting in reduced Ca^2+^ permeability of the NMDA receptor [21]. In general, Ca^2+^ signals originating from NMDA receptors are especially important for neural modulation, activating Ca^2+^-dependent intracellular signaling pathways [34, 35].

NMDA receptors have the important property of providing voltage-dependent Mg^2+^ blockade, and this ‘voltage-dependence’ enables the NMDA receptor to play a role as a coincident detector of activation between pre- and postsynaptic neurons [34–36]. This form of synapse is called a ‘Hebbian synapse’, and contributes to modulation of not only local synapses, but also the overall level of network function [37]. NMDA receptors may thus contribute to synchronization between populations of neurons. Taking the actions of postsynaptic GABA_B_ receptors into account, the function of oscillatory networks that require NMDA receptor activation is presumed to change according to the strength of GABA_B_ receptor activation.

We have previously reported that activity-dependent stable membrane potential oscillations at 8–10 Hz that require NMDA receptor activation are inducible in the somatosensory cortex of rats under caffeine application [38]. The area of somatosensory cortex we investigated in previous studies is considered to be the area receiving sensory inputs from the oral region [39–41], suggesting that the oral somatosensory cortex (OSC) may include neural oscillators. In addition, we revealed that the oscillatory origin repeatedly delivers oscillatory signals along horizontal layers, and that the oscillator is equipped in the upper layer of the OSC [42, 43]. The present study, therefore, focused on the OSC to investigate the role of GABA_B_ receptors in network oscillation.

Focusing on the synchronization of neural activities, Gu et al. reported that an increase in phase synchrony between two regions is dependent on NMDA receptors, and the NMDA receptor-dependent phase-locking of neural oscillation induces plastic changes in communication between regions [44]. In addition, NMDA receptors play a key role in controlling oscillation frequency [45]. Thus, the inhibitory network system accompanied by GABA_B_ receptors is hypothesized to act as a regulator of network oscillation when the network oscillation requires NMDA receptor activity. Interestingly, activation of NMDA receptors regulates surface expression of GABA_B_ receptors by enhancing the recycling of GABA_B_ subunits [21], suggesting that NMDA receptors and GABA_B_ receptors are interdependent. Conveniently, induction of the caffeine-assisted oscillation is activity dependent, requiring NMDA receptor activation during the induction process [38]. The present study examined whether and how slow synaptic modulation by GABA_B_ receptors regulates local network oscillation, focusing on NMDA receptors, using our experimental oscillation model.

## Methods

All experiments were approved by the Animal Care Committee of Tokushima Bunri University (approval no. KP22-83-4) and Kanazawa Medical University (approval no. 2012077). Experiments were performed in accordance with the Guidelines for the Ethical Use of Animals by the Physiological Society of Japan. All efforts were made to minimize both the number of animals and their suffering.

### Optical recording

The experimental method for slice preparation for the voltage-sensitive dye (VSD) recordings employed in this study is basically identical to the method previously described [46]. Briefly, coronal slices of rat forebrain including the somatosensory cortex were prepared. Wistar rats (28–35 days old) were decapitated under deep isoflurane anesthesia after perfusion with ice-cold artificial cerebrospinal fluid (aCSF; 124 mM NaCl, 2.5 mM KCl, 2 mM CaCl_2_, 2 mM MgSO_4_, 1.25 mM NaH_2_PO_4_, 26 mM NaHCO_3_, and 10 mM glucose; pH 7.4) bubbled with 95% O_2_/5% CO_2_ gas. Brains were quickly removed and cooled in aCSF. After cooling for 5 min, forebrain slices including the OSC were dissected and sliced into 400-μm transverse sections using a vibratome (Leica VT-1200S).

After 1 h of incubation, each slice was stained for 25 min with 100 μl of VSD solution containing 0.2 mM Di-4-ANEPPS (D-1199, Molecular Probes Inc. OR, USA) in 2.5% ethanol, 0.13% Cremophor EL (Sigma), 1.17% distilled water, 48.1% fetal bovine serum (Sigma), and 48.1% aCSF. After washing to remove the VSD, slices were incubated at 28 °C for at least 1 h, then left at room temperature before imaging by optical recording.

For optical recordings, the Plexiglas ring supporting each slice was placed in an immersion-type recording chamber [47]. Slices were continuously perfused with pre-warmed (31 °C) and oxygenated aCSF (bubbled with a 95% O_2_/5% CO_2_ gas mixture) at a rate of 1 ml/min. The high-speed imaging system provided spatial resolution of approximately 22 × 22 μm at the objective (96 pixels × 64 pixels resolution, MiCAM02, BrainVision, Inc., Tokyo, Japan). Optical signals referred to in the following sections represent signals filtered in spatial and temporal dimensions with a Gaussian kernel of 5 × 5 × 3 (horizontal × vertical × temporal).

To investigate evoked optical signals, a stimulating glass electrode filled with aCSF (1 MΩ) was placed in layer IV of the OSC. The duration and intensity of stimuli were 300 μs and 40–50 V, respectively. We analyzed optical signals offline using a procedure developed for IgorPro (WaveMetrics Inc, OR, USA). For additional details on the methods, see Tominaga et al. and Gusain et al. [46–49].

### Field potential recording

The experimental method for slice preparation for field potential recordings employed in this study was basically identical to the method previously described [38]. Wistar rats (25–29 days old) were used for electrophysiological recordings. Before starting these recordings, rats were decapitated under deep isoflurane anesthesia and the brain was quickly removed and placed in cold medium (2–4 °C) containing 124 mM NaCl, 3.3 mM KCl, 1.25 mM NaH_2_PO_4_, 1.3 mM MgSO_4_, 2 mM CaCl_2_, 26 mM NaHCO_3_, and 10 mM d-glucose and saturated with 95% O_2_/5% CO_2_. Brain slices (300–350 μm thick) including the OSC were prepared using a slicer. Once cut, slices were left at room temperature for at least 1 h before starting the recording session.

For field potential recordings, slices were placed in a submerged-type chamber set on the stage of an upright microscope (IMT-2; Olympus, Tokyo, Japan), and perfused with 30 °C medium at 5 ml/min. Micropipettes for field potential recordings were filled with 3 M NaCl and inserted into layer II/III in the OSC. To investigate evoked field potentials, a bipolar tungsten electrode was inserted into layer IV in the somatosensory cortex. The duration and intensity of stimuli were 80 μs and 250–350 μA, respectively. Synaptic responses were recorded using a bridge-equipped amplifier (Axoclamp-2B; Axon Instruments, Foster City, CA, USA), digitized using an AD converter (rate, 2.5–5 kHz, Digidata 1200; Axon Instruments) and stored on a personal computer for offline analysis. Precise waveform analysis was performed using Origin8 software (OriginLab Co., Northampton, MA, USA).

### Protocol for oscillation induction and procedure for recordings

According to the purposes of the study, caffeine (3 mM) (purchased from Wako Pure Chemical Industries, Osaka, Japan) was added to the medium. We have developed a protocol in which synchronized population oscillation of synaptic potentials at a frequency of 8–10 Hz is induced in slices of rat visual cortex [50–52], retrosplenial cortex [52, 53], endopiriform nucleus [54] and somatosensory cortex [38, 42, 43] bathed in caffeine-containing medium. Low-frequency stimulation at a frequency of 0.3 Hz was continued for approximately 30 min. When the frequency of stimulation was changed from 0.3 to 0.03 Hz, stable oscillation was induced. The concentration of extracellular Mg^2+^ is particularly important for inducing the oscillation, and we precisely investigated the contribution of Mg^2+^ at concentrations of 0.1 mM, 0.5 mM and 1.3 mM [42, 50, 55]. While the characteristics of oscillation in 0.5 mM Mg^2+^ and in 1.3 mM Mg^2+^ appear almost the same, the induction probability of oscillation in 0.5 mM Mg^2+^ is markedly higher. Therefore, to identify the precise location of the oscillatory origin, we initiated optical recordings in 0.5 mM Mg^2+^, whereas most recordings using field potential recording were performed in 1.3 mM Mg^2+^.

All synaptic responses for data analysis were elicited by electrical stimulation at a frequency of 0.03 Hz. Neural responses were recorded from the area of the somatosensory cortex, as described in the atlas by Paxinos and Watson [39]. The delineation of area borders was based on the descriptions by Swanson [40] and Zilles in Paxinos’ “The Rat Nervous System” [39–41]. According to the purposes of the experiment, the following chemicals were applied to the extracellular medium: caffeine, 3 mM; d-2-Amino-5-phosphonovaleric acid (d-AP5), 10 μM; 6-cyano-7-nitroquinoxaline-2,3-dione (CNQX), 20 μM; baclofen, 10 μM, 1 μM, or 0.1 μM; and thapsigargin, 15 μM.

## Results

### Oscillatory origin and identification of receptors involved in oscillatory events: applying the optical recording method

**Fig. 1:**
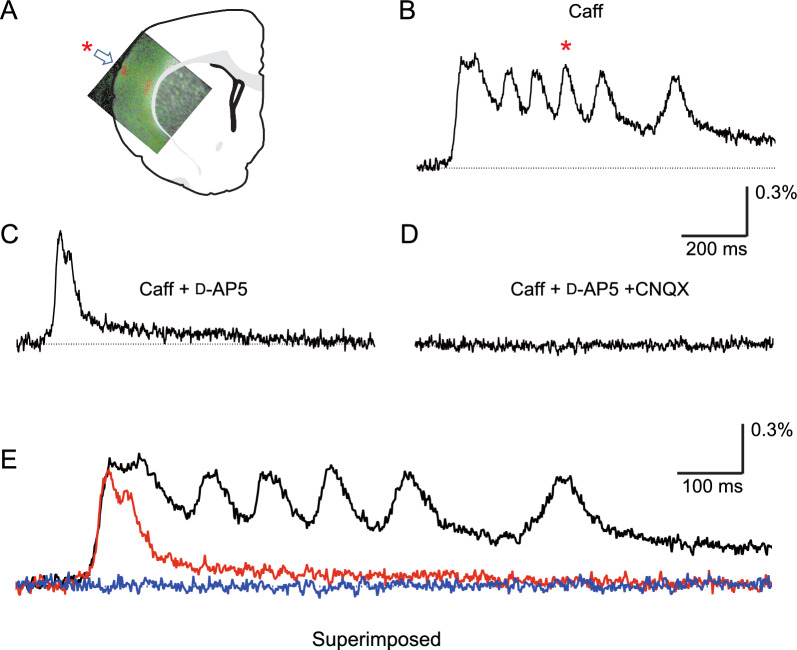
Pharmacological analysis of caffeine-assisted oscillation elicited by intra-cortical stimulation in the OSC, focusing on NMDA receptors. An optical recording method was applied. All recordings were performed in caffeine-containing medium. **A** Optical image acquired at a time latency of 337.0 ms superimposed on the slice illustration. The image includes a high-activity area during one course of oscillation (as shown in **B**). Note that a high-activity area that emerged repeatedly is considered the origin of oscillatory activities. Thus, the location of oscillatory origin was confirmed to be in the upper layer of the somatosensory cortex, as indicated by the arrow. **B** Time-course responses of the optical signal obtained at the oscillatory origin (as shown in **A**). Note that the local area shown in A with the asterisk generated the wave with the asterisk in **B**. **C** Time-course response after application of d-AP5 in caffeine-containing medium. Note that the oscillatory phase completely disappeared, but the initial wave did not. The initial slope and peak amplitude of the initial wave were almost the same as those before application of d-AP5. **D** Time-course response after further application of CNQX in medium containing both caffeine and d-AP5. Note that the initial wave completely disappeared. **E** The 3 time-course responses shown in **B**–**D** are superimposed, and the temporal axis is expanded

First, we performed optical recordings to identify the site of oscillatory origin (Fig. 1A). The site of oscillatory origin was confirmed to be within the OSC (Fig. 1A), and the time-course response of optical signals obtained at the oscillatory origin are shown in Fig. 1B. A later oscillatory phase was NMDA receptor dependent, since application of d-AP5 completely abolished later oscillatory waves, but not the initial wave (Fig. 1C). The residual initial wave was non-NMDA receptor dependent, since additional application of CNQX completely abolished the initial wave (Fig. 1D). In addition, a notable property was that the initial slope and peak amplitude of the residual initial wave were unaffected by d-AP5 application (Fig. 1B, C, and E).

**Fig. 2:**
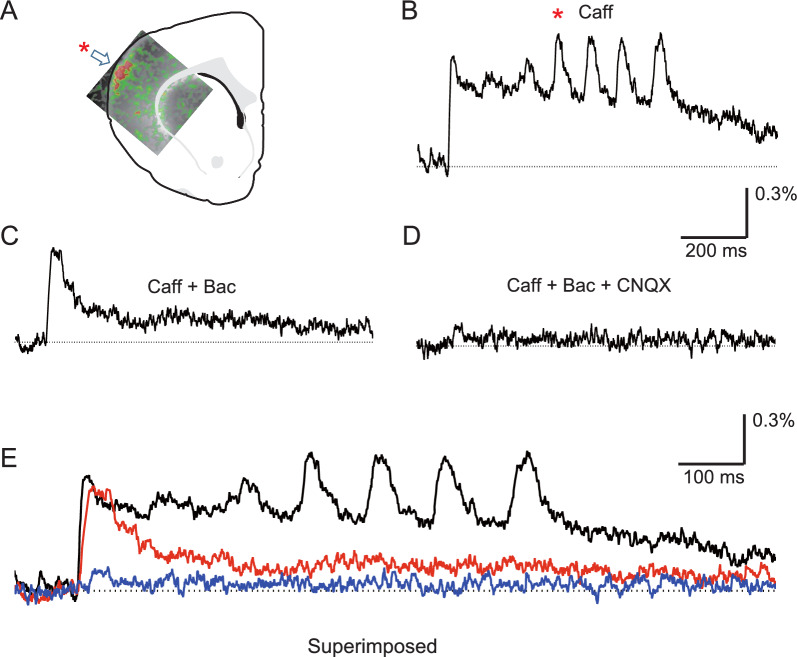
Pharmacological analysis of caffeine-assisted oscillation elicited by intra-cortical stimulation in the OSC, focusing on GABA_B_ receptors. An optical recording method was applied. All recordings were performed in caffeine-containing medium. **A** Optical image acquired at a time latency of 310.8 ms is superimposed on the slice illustration. The image includes a high-activity area during one course of oscillation (as shown in **B**). Note that the high-activity area that emerged repeatedly is considered the origin of oscillatory activities. The location of oscillatory origin was thus confirmed to be in the upper layer of the somatosensory cortex, as indicated by the indicated by the arrow. **B** One time-course response of the optical signal obtained at the oscillatory origin (as shown in **A**). Note that the local area shown in A with the asterisk generated the wave with the asterisk in **B**. **C** Time-course response after application of baclofen in caffeine-containing medium. Note that the oscillatory phase completely disappeared, but the initial phase did not. The initial slope and peak amplitude of the initial wave were slightly decreased as compared with those before the application of baclofen, and slight depolarization continued during the later phase. **D** Time-course response after further application of CNQX in medium containing both caffeine and baclofen. Note that most of the initial wave disappeared. **E** The 3 time-course responses shown in **B**–**D** are superimposed, and the temporal axis is expanded

In the next experiments, we investigated whether activation of GABA_B_ receptors affected caffeine-assisted oscillation. Figure 2A shows the location of the oscillatory origin in the OSC, and Fig. 2B shows the time-course response of optical signals obtained at the oscillatory origin. We then confirmed that application of baclofen (10 μM) abolished later oscillatory waves, but not the initial wave (Fig. 2C). Interestingly, the NMDA receptor-dependent oscillatory phase was markedly blocked by the application of baclofen, suggesting that activation of GABA_B_ receptors affects the NMDA receptor activity-dependent phase (Figs. 1C, 2C). The residual initial wave was mainly non-NMDA receptor dependent, since additional application of CNQX almost abolished the initial wave (Fig. 1D). In this case, the initial slope and peak amplitude of the residual initial wave were slightly reduced by the application of baclofen (Fig. 2E), showing that the initial wave involving non-NMDA receptors may be slightly affected by GABA_B_ receptor activation.

### Actions of GABAB receptor on oscillatory events: applying a field potential recording method

**Fig. 3:**
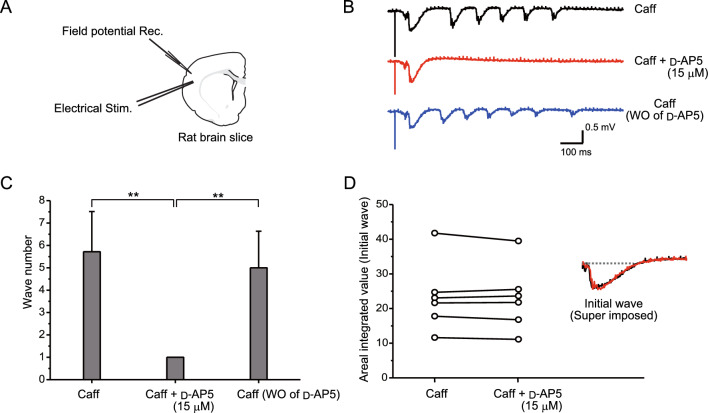
Analysis of the initial wave and later oscillatory phase of caffeine-assisted oscillation, focusing on NMDA receptors. A field potential recording method was applied. **A** A glass micropipette for field potential recording and an electrode for stimulation are shown in a brain slice illustration. Field potentials were recorded from layer II/III, and electrical stimulation was delivered to layer IV in the OSC. **B** Waveforms obtained by field potential recording. Stable oscillation was generated in the caffeine-containing medium (top). Application of d-AP5 blocked the oscillatory phase, but not the initial phase (middle). After washout of d-AP5, the oscillatory phase reappeared (bottom). **C** Averages of wavelet number before and during d-AP5 application to caffeine-containing medium, and after washout of d-AP5. **D** Areal integrated values of the initial wave before and after application of d-AP5. Representative waveforms before and during d-AP5 application are superimposed and shown in the graph. Note that application of d-AP5 has little effect on the initial wave. Asterisks (*P < 0.005; **P < 0.001) indicate significant statistical differences

A glass micro-electrode for field potential recording was placed in layer II/III of the OSC, the site of oscillatory origin (Figs. 1, 2, 3A). Figure 3B shows the waveforms of field potentials under different conditions. Marked oscillation was induced under application of caffeine (Fig. 3B, top). After inducing stable oscillation, application of d-AP5 completely abolished the later oscillatory phase (Fig. 3B, middle), and the abolished phase reappeared after washout of d-AP5 (Fig. 3B, bottom), showing that the later oscillatory phase was NMDA receptor dependent. Figure 3C shows the effect of d-AP5 application on the size of oscillation under caffeine application, using averaged wavelet numbers. One-way analysis of variance (ANOVA) (Origin 8; OriginLab Co., USA) was used to evaluate the statistical differences and revealed significant differences (*n* = 5, *F* = 22.96; *P* = 1.1E−5). Scheffe’s post hoc test was then used to detect differences between pharmacological manipulations. Oscillation sizes after the application of d-AP5 were reduced compared with those before the application of d-AP5 (*n* = 5, *F* = 19.76; *P* = 2.87E−5), and oscillation size after washout of d-AP5 was recovered compared with those after the application of d-AP5 (*n* = 5, *F* = 14.23; *P* = 1.97E−4), suggesting that application of d-AP5 actually reduced oscillation size.

We then investigated whether the initial wave is affected by d-AP5 application. We calculated the area surrounded by the lines of fluctuation and the baseline before the emergence of a response. Representative examples are shown in Fig. 3D. Areal integrated values of initial waves were plotted before and after application of d-AP5, finding no significant differences (paired *t*-test, *n* = 6; *P* = 0.463) (Fig. 3D). In more detail, the initial slope and peak amplitude were unaffected by the application of d-AP5, showing that there was almost no NMDA receptor component in the initial wave.

**Fig. 4:**
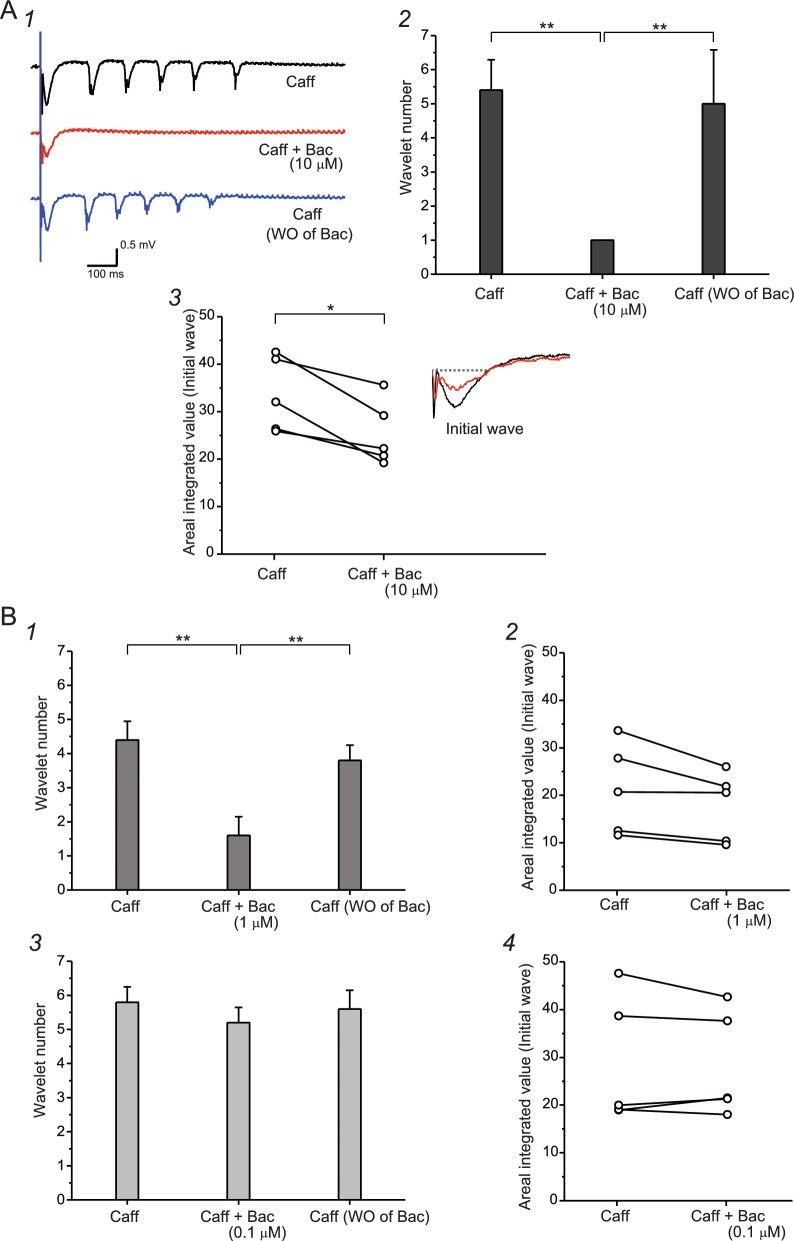
Analysis of the initial wave and later oscillatory phase of caffeine-assisted oscillation, focusing on GABA_B_ receptors. Field potential recording was performed, and electrical stimulation was delivered in the same manner shown in Fig. 3A. **A1** Waveforms obtained by field potential recording. Stable oscillation was generated in the caffeine-containing medium (top). Application of 10 μM baclofen blocked the oscillatory phase, but not the initial phase (middle). After washout of baclofen, the oscillatory phase reappeared (bottom). **A2** Averages of wavelet number before and during baclofen application to caffeine-containing medium, and after washout of baclofen. **A3** Areal integrated values of the initial wave before and after application of baclofen. Representative waveforms before and during baclofen application are superimposed and shown in the graph. Note that application of baclofen clearly decreases initial wave size. **B1** Averages of wavelet number before and during baclofen (1 μM) application to caffeine-containing medium, and after washout of baclofen. **B2** Areal integrated values of initial wave before and after application of baclofen. Note that application of baclofen tends to decrease the initial wave size. **B3** Averages of wavelet number before and during baclofen (0.1 μM) application to caffeine-containing medium, and after washout of baclofen. **B4** Areal integrated values of initial wave before and after application of baclofen. Note that application of low-concentration baclofen does not seem to affect initial wave size. Asterisks (*P < 0.005; **P < 0.001) indicate significant statistical differences

In the next experiments, we investigated how the activation of GABA_B_ receptors affected caffeine-assisted oscillation. Figure 4A1 shows the waveforms of field potentials. Marked oscillation was induced under caffeine application (Fig. 4A1, top). After inducing stable oscillation, application of baclofen (10 μM) completely abolished later oscillatory waves (Fig. 4A1, middle), and the abolished phase reappeared after baclofen washout (Fig. 4A1, bottom), showing that the activation of GABA_B_ receptors occurs in the later oscillatory phase. Figure 4A2 shows the effects of baclofen application on the size of the oscillation under caffeine application, using averaged wavelet numbers. One-way ANOVA (Origin 8) was used to evaluate statistical differences, revealing significant differences (*n* = 5, *F* = 26.91; *P* = 3.67E−5). Scheffe’s post hoc test was then used to detect differences between pharmacological manipulations. Oscillation sizes after baclofen application were reduced compared with those before baclofen application (*n* = 5, *F* = 22.0; *P* = 9.68E−5), and oscillation size after baclofen washout was recovered compared with that d-AP5 application (*n* = 5, *F* = 18.18; *P* = 2.33E−4), suggesting that the application of baclofen actually reduced oscillation size. We then investigated whether the initial wave was affected by baclofen application. We calculated the area surrounded by the lines of fluctuation and the baseline before the emergence of response. Representative examples are shown in Fig. 4A3. Areal integrated values of initial waves were plotted before and after baclofen application, showing a significant difference between these waves (paired-*t* test, *n* = 5; *P* = 0.0158) (Fig. 4A3), showing that a component affected by GABA_B_ receptor activation was included in the initial wave.

In the same manner, we investigated whether these actions of baclofen are dependent on the concentration of baclofen. Figure 4B1 shows the effects of baclofen application on the size of oscillation under application of a lower concentration of baclofen (1 μM), using averaged wavelet numbers. One-way ANOVA (Origin 8) was used to statistically evaluate comparisons, revealing significant differences (*n* = 5, *F* = 40.75; *P* = 4.47E−6). Scheffe’s post hoc test was then used to detect differences between pharmacological manipulations. In the case of 1 μM baclofen, oscillation sizes after baclofen application were reduced compared with those before application (*n* = 5; *F* = 36.75; *P* = 7.64E−6). Oscillation size after baclofen washout was recovered compared with those with baclofen application (*n* = 5, *F* = 22.69; *P* = 8.37E−4) (Fig. 4B1). However, in the case of 1 μM baclofen, no significant differences in the magnitude of the initial wave were evident between before and after baclofen application (paired-*t* test, *n* = 5; *P* = 0.05331) (Fig. 4B2). Next, in case of 0.1 μM baclofen, no significant differences were evident in oscillation size (one-way ANOVA, *n* = 5, *F* = 0.928; *P* = 0.417) (Fig. 4B3). In this case, no significant differences in the magnitude of the initial wave were identified between before and after baclofen application (paired-*t* test, *n* = 5; *P* = 0.649) (Fig. 4B4).

**Fig. 5:**
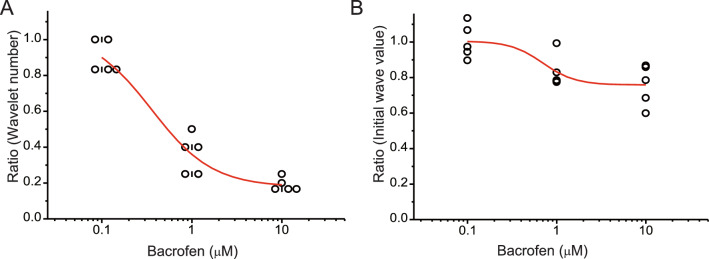
Dose-dependent effects of baclofen. **A** Ratios of wavelet number before and after application of d-AP5 are plotted according to concentration of baclofen, followed by sigmoid curve fitting. Note that attenuation of oscillation by baclofen appears strongly dose dependent (*R*^2^ = 0.925). **B** Ratios of areal values of the initial wave before and after application of baclofen are plotted according to the concentration of baclofen, followed by sigmoid curve fitting. Note that attenuation of initial wave size by baclofen appears weakly dose dependent (*R*^2^ = 0.437)

The above-mentioned results suggest that the effects of baclofen on caffeine-assisted oscillation depended on the concentration of baclofen. First, we investigated the relationship between the ratio of wavelet number (after/before) and baclofen concentration. Sigmoid fitting revealed that oscillation size was markedly dependent on the dose of baclofen (*F* = 156.29, *R*^2^ = 0.925, *P* = 2.68E−9) (Fig. 5A). Second, we investigated the relationship between the ratio of the initial wave magnitude (after/before) and baclofen concentration. Sigmoid fitting revealed that the magnitude of the initial wave was dependent on the dose of baclofen (*F* = 250.9, *R*^2^ = 0.437, *P* = 2.08E−10), but this dose dependence was weak (Fig. 5B). Thus, the effects of baclofen on the size of caffeine-assisted oscillation and on the magnitude of the initial wave are dependent on the dose of baclofen. These results suggest that the functions of oscillatory networks that require NMDA receptor activation are changeable according to the strength of GABA_B_ receptor activation.

### Postsynaptic intracellular Ca^2+^ and NMDA receptor-assisted oscillation

**Fig. 6:**
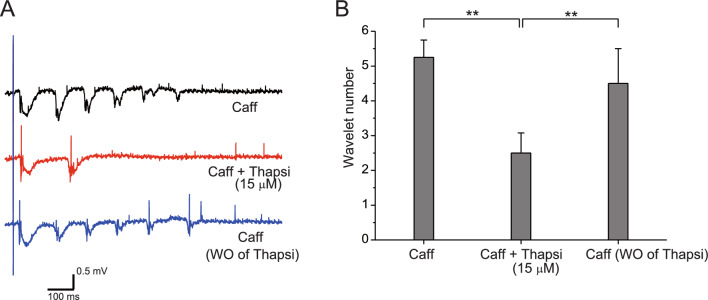
Attenuation of caffeine-assisted oscillation by depletion of intracellular Ca^2+^ stores. Field potential recordings were performed, and electrical stimulation was delivered in the same way as shown in Fig. 3A. **A** Waveforms obtained by field potential recording. Stable oscillation was generated in the caffeine-containing medium (top). Application of 15 μM thapsigargin blocked the later oscillatory phase, but the initial and second waves remained (middle). After washout of baclofen, the oscillatory phase reappeared (bottom). **B** Averages of wavelet number before and during thapsigargin application to caffeine-containing medium, and after washout of thapsigargin. Asterisks (**P < 0.001) indicate significant statistical differences

GABA_B_ receptors may regulate postsynaptic Ca^2+^ dynamics by way of NMDA receptors. If that is indeed the case, Ca^2+^-induced Ca^2+^ release (CICR) may be affected, resulting in the attenuation of oscillatory activities. We then investigated how depletion of intracellular Ca^2+^ stores affects NMDA receptor-dependent oscillatory activities. Figure 6A shows the waveforms of field potentials under different conditions. Marked oscillation was induced under caffeine application (Fig. 6A, top). After inducing stable oscillation, application of thapsigargin completely abolished the later oscillatory phase, but the initial and second waves remained (Fig. 6A, middle), and the abolished phase reappeared after thapsigargin washout (Fig. 6A, bottom), showing that appearance of the later oscillatory phase required Ca^2+^ release from intracellular Ca^2+^ stores.

Figure 6B shows the effect of applying thapsigargin on the size of oscillation under caffeine application, using averaged wavelet numbers. One-way ANOVA (Origin 8) was used to evaluate statistical differences, revealing significant differences (*n* = 4, *F* = 26.91, *P* = 3.67E−5). Scheffe’s post hoc test was then used to detect differences between pharmacological manipulations. Oscillation sizes after thapsigargin application were reduced compared with those before thapsigargin application (*n* = 4, *F* = 22.0; *P* = 9.68E−5), and oscillation size after baclofen washout was recovered compared with those before thapsigargin application (*n* = 4, *F* = 18.18; *P* = 2.33E−4), suggesting that the application of thapsigargin actually reduced oscillation size.

## Discussion

### Summary of present results

**Fig. 7:**
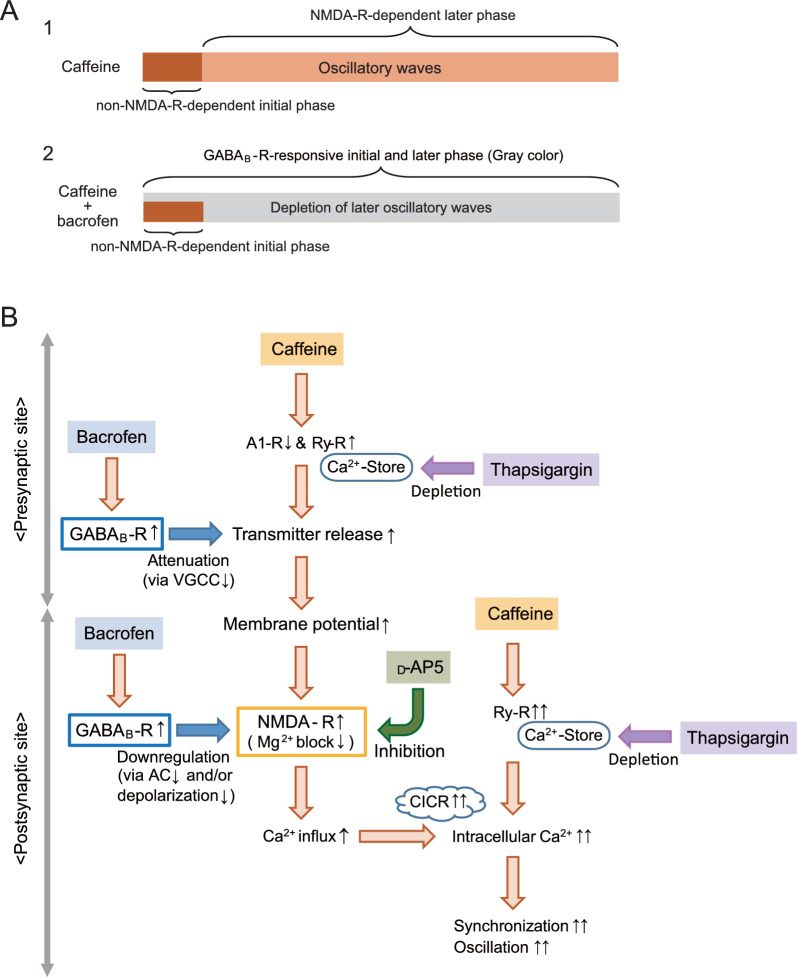
Summary of results. **A1** Caffeine-assisted oscillation was composed comprised an initial phase that is non-NMDA receptor dependent and a later oscillatory phase that is NMDA receptor dependent. **A2** Application of baclofen attenuated the initial phase, and abolished the later oscillatory phase. Gray color area shows GABA_B_ receptor-responsive components. Residual initial phase was non-NMDA receptor dependent. **B** Presumed downstream of GABA_B_ receptor and NMDA receptor based on the present experiments. Application of caffeine induced oscillation via increase in NMDA receptor activity and CICR. Application of baclofen attenuated transmitter release at presynaptic site, and inhibited oscillatory phase via downregulation of NMDA receptor at postsynaptic site. Pharmacological actions of d-AP5 and thapsigargin are also illustrated. *A1-R* Adenosine A1 receptor, *Ry-R* ryanodine receptor, *AC* adenylyl cyclase

Caffeine-assisted oscillation was comprised an initial non-NMDA receptor-dependent phase and a later NMDA receptor-dependent oscillatory phase (Figs. 1, 3, 7A1). GABA_B_ receptor agonist, baclofen, markedly abolished the NMDA receptor dependent later oscillatory phase, whereas, slightly attenuated the non-NMDA receptor-dependent initial phase (Figs. 2, 4, 7A2). The effects of baclofen on caffeine-assisted oscillation depended on the concentration of baclofen, suggesting that the functions of oscillatory networks that require NMDA receptor activation are changeable according to the strength of GABA_B_ receptor activation. In addition, depletion of intracellular Ca^2+^ store by thapsigargin markedly abolished the NMDA receptor dependent later oscillatory phase (Fig. 6), suggesting that CICR may be deeply involved in induction of caffeine-assisted oscillation. Thus, Ca^2+^ signals may play important roles in controlling by GABAB receptor. Presumed downstream of GABA_B_ receptor and NMDA receptor based on the present experiments is illustrated in Fig. 7B. In the following paragraphs, we will discuss how GABA_B_ receptor regulates oscillatory activities at presynaptic and postsynaptic sites.

### Contribution of inhibitory network on regulation of membrane potential oscillation

GABA_A_ receptors are coupled with Cl^−^ channels and mediate fast synaptic inhibition within the excitatory network. GABA_A_ receptor-mediated inhibition enables the generation of synchronized excitation between populations of excitatory neurons [11, 19, 56], and different durations of inhibition could be associated with the production of different frequencies of oscillation [11]. Within neural networks, interneurons play an important role in allowing synchronization. The induction and maintenance of synchronized network oscillations may depend not only on GABA_A_ receptor-mediated inhibition, but also on the recruitment of interneuron firing by excitation of the glutamatergic network [11]. In the present study, application of caffeine increased excitatory networks, particularly NMDA receptor-mediated synaptic transmission. One of the major pharmacological actions of caffeine is blockade of GABA_A_ receptors at high doses [57]. Since we used high doses of caffeine (3 mM), GABA_A_ receptor-mediated inhibition seems unlikely to have contributed to the generation of synchronization.

Unlike GABA_A_ receptor-mediated fast synaptic inhibition, GABA_B_ receptor-mediated slow synaptic inhibition is achieved by way of intracellular mechanisms. However, the contribution of GABA_B_ receptors to network oscillations is less understood. Recently, the roles of interneurons in synchronization, focusing on GABA_B_ receptors, have been demonstrated. In the case of hippocampal theta and gamma oscillations induced by carbachol application, the activation of GABA_B_ receptors following baclofen application diminished the generation of oscillatory waves [25]. Under those circumstances, activation of presynaptic GABA_B_ receptors uncoupled somatostatin-expressing interneurons from oscillatory networks, resulting in the disappearance of oscillatory activity. Interneurons were thus revealed to play a role in the generation of synchronization, and presynaptic GABA_B_ receptors regulate network oscillation by acting on interneurons. Somatostatin-expressing interneurons also exist in the somatosensory cortex, controlling cortical information processing [58, 59]. Further, GABA_B_ receptors are expressed on somatostatin interneurons in the somatosensory cortex, and modulate network activities [60]. Presynaptic GABA_B_ receptors might, therefore, play a role in achieving synchronization by way of interneurons in the somatosensory cortex. In the present study, however, predicting the roles of interneurons in achieving synchronization is difficult, based on the present data. In the case of caffeine-assisted oscillation, further experiments are required, focusing on interneurons and presynaptic GABA_B_ receptors.

### Intracellular Ca^2+^ signal and oscillation

In general, caffeine acts on ryanodine receptors expressed at intracellular Ca^2+^ store, resulting in enhancement of release of stored Ca^2+^ [57]. In addition, an important characteristic of NMDA receptor is Ca^2+^ permeability [34–36]. Therefore, Ca^2+^ dynamics may underlie the caffeine-assisted oscillation, and should be taken into account in considering actions of GABA_B_ receptor.

A recent study demonstrated that GABA_B_ receptors inhibit the Ca^2+^ permeability of NMDA receptors to decrease Ca^2+^ signals in postsynaptic spines [31]. The underlying mechanisms are as follows. GABA_B_ receptors are G-protein coupled receptors, and once the GABA_B_ receptor is activated, the AC is inhibited. This results in a reduced cAMP level, inducing the attenuation of NMDA receptor activity through decreases in protein kinase A (PKA) [33]. Thus, activation of GABA_B_ receptors selectively modulates Ca^2+^ permeability of the NMDA receptor. An important function of the NMDA receptor is that Ca^2+^ influx through the NMDA receptor triggers CICR, resulting in the induction of various synaptic changes. When NMDA receptor activity is attenuated by baclofen, intracellular Ca^2+^ release from Ca^2+^ stores may be reduced.

Regarding Ca^2+^ signals, we have already reported that depletion of intracellular Ca^2+^ prevented the induction of caffeine-assisted oscillations in the visual cortex [38, 42, 43]. In addition, CICR is required for the induction of caffeine-assisted oscillation [50, 55]. Thus, with synchronized oscillation that is related to NMDA receptor activity through downstream GABA_B_ receptors and NMDA receptors, Ca^2+^ signals may play important roles in the generation and regulation of synchronized oscillation.

In the present study, we confirmed that depletion of intracellular Ca^2+^ stores by thapsigargin attenuated oscillation size, particularly for the later oscillatory phase (Fig. 6). In addition, our previous study demonstrated that co-application of caffeine and ryanodine prevented induction of caffeine-assisted oscillations [42]. These results led us to the strong certainty that GABA_B_ receptors can control CICR via the modulation of NMDA receptor function.

NMDA receptors are also expressed at presynaptic terminals, and Ca^2+^ influx through NMDA receptors contributes to synaptic function. However, increases in Ca^2+^ concentration at presynaptic terminals for transmitter release are predominantly caused by the opening of voltage-gated Ca^2+^ channels [61, 62]. Indeed, functional Ca^2+^ stores exist in synaptic terminals and the stored Ca^2+^ can be released either spontaneously or by Ca^2+^ influx through VGCCs. Actually, inhibition of evoked neurotransmitter release by GABA_B_ receptors is generally associated with inhibition of VGCCs via released Gβγ, which inhibits Ca^2+^ influx at the synaptic terminals [26]. Conversely, postsynaptic GABA_B_ receptor activation robustly suppresses NMDA receptor Ca^2+^ signals, independent of any effects on presynaptic release [30]. Taking these findings into account, Ca^2+^ depletion of intracellular Ca^2+^ stores with the administration of thapsigargin may predominantly influence postsynaptic function under the present conditions. Indeed, Ca^2+^ influx through VGCCs also triggers CICR [63], but, in the case of caffeine-assisted oscillation, Ca^2+^ influx through NMDA receptors seems to represent the main cause of CICR, since the later oscillatory phase is completely contingent on NMDA receptor activation (Fig. 1).

### Mechanisms underlying interactions between NMDA receptors and GABAB receptors

NMDA receptors are expressed at both the postsynaptic dendritic spine and the extra-synaptic membrane, as synaptic NMDA receptors and extra-synaptic NMDA receptors, respectively. Since the GluN2B subunit is present in extra-synaptic NMDA receptors, voltage-dependent Mg^2+^ block at the NMDA receptor is reduced [64–66], and one characteristic of the extra-synaptic NMDA receptor is the low threshold of Ca^2+^ entry. Hyper-excitation of the extra-synaptic NMDA receptor may, therefore, induce a pathological condition [67]. An important role of synaptic NMDA receptors is co-incidence detector between pre- and post-synaptic activity, in which voltage-dependent Mg^2+^ block enables the induction of synaptic plasticity [34, 37, 68, 69]. In terms of the local network level of caffeine-assisted oscillation, we previously reported that repetitive activation of NMDA receptors enhances non-NMDA receptor activity in the visual cortex, which seems to represent one of local network synaptic plasticity [52, 70]. This is consistent with a previous study in which Lu et al. demonstrated that selective activation of synaptic NMDA receptors induced insertion of new non-NMDA receptors onto the membranes of excitatory synapses [71]. Therefore, during the induction process for caffeine-assisted oscillation, synaptic NMDA receptor activity may be the main form enhanced, not extra-synaptic NMDA receptor activity.

Focusing on adenosine receptors in the neocortex, A1 receptors, as one subtype of adenosine receptor, negatively influence transmitter release from presynaptic terminals by inhibiting AC [57]. Caffeine is a nonspecific adenosine receptor antagonist, but acts predominantly on the A1 receptors of glutamatergic synapses in the cortical regions and positively influences presynaptic transmitter release via the blockade of A1 receptors [72, 73]. Considering these pharmacological actions, synaptic NMDA receptors rather than extra-synaptic NMDA receptors appear predominantly engaged in the emergence of caffeine-assisted oscillation.

The GABA_B_ receptor is a heterometric G-protein-coupled receptor, and is divided into GABA_B1_ and GABA_B2_ classes. The GABA_B1_ subunit contains the ligand-binding domain, while GABA_B2_ couples to the G-protein to downregulate AC [17, 32, 74, 75]. GABA_B_ receptors are abundantly expressed on the plasma membrane of glutamatergic synapses [76–79]. In particular, GABA_B1_ and GABA_B2_ co-localize mostly at postsynaptic sites [80]. Kantamneni et al. reported that selective activation of synaptic NMDA receptors increases the surface expression of GABA_B1_ and GABA_B2_ by enhancing the recycling of GABA_B_ subunits [21]. In contrast, global activation of synaptic and extra-synaptic NMDA receptors causes the loss of surface GABA_B_ receptors due to endosomal trafficking [15]. In case of caffeine-assisted oscillation, synaptic NMDA receptor activity may be predominantly activated, as mentioned above. Consequently, GABA_B_ receptors comprising GABA_B1_ and GABA_B2_ subunits are expressed at postsynaptic spines. Baclofen would thus presumably dramatically depress the NMDA receptor-dependent oscillatory phase.

Under the ‘caffeine + baclofen’ condition (Fig. 2C), membrane potential during the later phase was markedly decreased compared with the ‘caffeine-only’ condition (Fig. 2B). This suggests the following possibility. Depletion of the later oscillatory phase may be caused by voltage-dependent Mg^2+^ block, but not via intracellular mechanisms that involve the down-regulation of AC. If that is the case, inward current through the NMDA receptor is reduced by enhancing voltage-dependent Mg^2+^ block due to insufficient depolarization caused by the inhibitory effect of the GABA_B_ receptor, such as opening K^+^ channel (e.g., GIRKs) and inducing a decrease in Ca^2+^ influx through NMDA receptors. As a result, caffeine-assisted oscillations that require an increase in intracellular Ca^2+^ might be attenuated.

Regarding this, notable findings were demonstrated. Chalifoux et al. reported that GABA_B_ receptors selectively modulate the Ca^2+^ permeability of NMDA receptors, and changes in ‘Mg^2+^ block’ cannot explain these effects of GABA_B_ receptors on the Ca^2+^ signaling of NMDA receptors [30]. This finding provides the idea that regulation of the oscillatory phase is mainly caused by an intracellular AC pathway, but not by voltage-dependent Mg^2+^ block. However, the reason the residual sustained slow depolarization after baclofen application was much smaller remains unclear (Fig. 2E). Sustained slow depolarization must be produced by NMDA receptor component, since no non-NMDA receptor-dependent component is included in the later phase, suggesting that inward current through the NMDA receptor remains. Indeed, the ratio of Ca^2+^ current against total current is difficult to detect, but intracellular Ca^2+^ dynamics must hold the key to answering this question. To elucidate the precise mechanisms by which GABA_B_ receptors regulate NMDA receptor-dependent oscillation, future studies should focus on the spatiotemporal dynamics of intracellular Ca^2+^.

### Regarding the OSC

Finally, the present study focused attention on the OSC, demonstrating that one role of GABA_B_ receptors is the regulation of network oscillation. Indeed, multiple tooth-losses during the developmental period prevent the emergence of caffeine-assisted oscillation in the OSC [81]. Under those conditions, the conduction of various kinds of sensory information from the peripheral oral region to the OSC may be decreased. As mentioned earlier, we have previously reported that the medial and lateral secondary visual cortex [51, 52, 70, 82], retrosplenial cortex [42, 52] and endopiriform nucleus [54] equip neural oscillators. These areas that include reciprocal connections are considered to have associative properties [83, 84]. The OSC may thus likewise represent an association area. Considering that activation of NMDA receptors regulates GABA_B_ receptor surface expression by enhancing the recycling of GABA_B_ subunits [21], the OSC may have a protection system against hyper-excitation that involves GABA_B_ receptors.

## Conclusion

This study investigated the role of GABA_B_ receptors in the regulation of network oscillation, focusing on NMDA receptors. Increased neural activities with the application of caffeine generated 8- to 10-Hz oscillations in the OSC. These caffeine-assisted oscillatory events comprised a non-NMDA receptor-dependent initial phase and a later NMDA receptor-dependent oscillatory phase. The later oscillatory phase occupied most of one course of an oscillatory event. Emergence of the later oscillatory phase was intracellular Ca^2+^-dependent, and application of baclofen, a GABA_B_ receptor agonist, markedly abolished the later oscillatory phase, but not the initial phase. Since intracellular signal pathways are operated by GABA_B_ receptors, NMDA receptor function may be modulated by the metabotropic actions of GABA_B_ receptors, resulting in decreased oscillatory activities. Thus, the local network in the OSC includes a down-regulatory system accompanied by GABA_B_ receptors.

## Data Availability

The datasets generated during and/or analyzed during the current study are available from the corresponding author on reasonable request.

## Abbreviations

aCSF: Artificial cerebrospinal fluid
ANOVA: Analysis of variance
cAMP: Cyclic adenosine monophosphate
CNQX: 6-Cyano-7-nitroquinoxaline-2,3-dione
AP5: (2*R*)-Amino-5-phosphonovaleric acid
GABA: γ-Aminobutyric acid
NMDA: *N*-Methyl-d-aspartate
PKA: Protein kinase A
VSD: Voltage-sensitive dye
VGCC: Voltage-gated Ca^2+^ channel
GIRK: G-protein-activated inwardly rectifying potassium channel

## References

[1] D’Souza RD, Wang Q, Ji W, Meier AM, Kennedy H, Knoblauch K, Burkhaler A (2022). Hierarchical and nonhierarchical features of the mouse visual cortical work. Nat Commun.

[2] Semedo JD, Jasper AI, Zandvakili A, Krishner A, Machens CK, Kohn A, Yu BM (2022). Feedforward and feedback interactions between visual cortical areas use different population activity patterns. Nat Commun.

[3] Shao Z, Burkhalter A (1996). Different balance of excitation and inhibition in forward and feedback circuits of rat visual cortex. J Neurosci.

[4] Mejias JF, Murray JD, Kennedy H, Wang W-J (2016). Feedforward and feedback frequency-dependent interactions in a large-scale laminar network of the primate cortex. Sci Adv.

[5] Buzáki G, Watson BO (2012). Brain rhythms and neural syntax: implications for efficient coding of cognitive content and neuropsychiatric disease. Dialog Clini Neurosci.

[6] Chadwick A, van Rossum MCW, Nolan MF (2015). Independent theta phase coding accounts for CA1 population sequences and enables flexible remapping. Elife.

[7] Singer W (2018). Neural oscillations: unavoidable and useful?. Eur J Neurosci.

[8] Barbero-Castillo A, Mateos-Aparicio P, Dalla Porta L, Camassa A, Perez-Mendez L, Sanchez-Vives MV (2021). Impact of GABA_A_ and GABA_B_ inhibition on cortical dynamics and perturbational complexity during synchronous and desynchronized states. J Neurosci.

[9] Lourenço J, De Stasi AM, Deleuze C, Bigot M, Pazienti A, Aguirre A, Giugliano M, Ostojic S, Bacci A (2020). Modulation of coordinated activity across cortical layers by plasticity of inhibitory synapses. Cell Rep.

[10] Sase T, Katori Y, Komuro M, Aihara K (2017). Bifurcation analysis on phase-amplitude cross-frequency coupling in neural networks with dynamic synapses. Front Comp Neurosci.

[11] Gonzalez-Burgos G, Lewis DA (2008). GABA neurons and mechanisms of network oscillations: implications for understanding cortical dysfunction in schizophrenia. Schizoph Bull.

[12] Whittington MA, Traub RD, Kopell N, Ermentrout B, Buhl EH (2000). Inhibition-based rhythms: experimental and mathematical observations on network dynamics. Int J Psychophysiol.

[13] Nicoll RA, Malenka RC, Kauer JA (1990). Functional comparison of neurotransmitter receptor subtypes in mammalian central nervous system. Physiol Rev.

[14] Connors BW, Malenka RC, Silva LR (1988). Two inhibitory postsynaptic potentials, and GABA_A_ and GABA_B_ receptor-mediated responses in neocortex of rat. J Physiol.

[15] Guetig N, Aziz SA, Holbro N, Turecek R, Rose T, Seddik R, Gassmann M, Moes S, Jenoe P, Ortner TG, Casanova E, Bettler B (2010). NMDA receptor-dependent GABA_B_ receptor internalization via CaMKII phosphorylation of serine 867 in GABAB1. Proc Nat Acad Sci USA.

[16] Bowery NG (1993). GABAB receptor pharmacology. Annu Rev Pharmacol Toxicol.

[17] Cryan JF, Kaupmann K (2005). Don’t worry ‘B’ happy!: a role for GABA_B_ receptors in anxiety and depression. Trends Pharmacol Sci.

[18] Lüscher C, Jan LY, Stoffel M, Malenka RC, Nicol RA (1997). G protein-coupled inwardly rectifying K^+^ channels (GIRKs) mediate postsynaptic but not presynaptic transmitter actions in hippocampal neurons. Neuron.

[19] Robbins MJ, Calver AR, Filippov AK, Hirst WD, Russel RB, Wood MD, Nasir S, Couve A, Brown DA, Moss SJ, Pangalos MN (2001). GABA_B2_ is essential for G-protein coupling of the GABA_B_ receptor heterodimer. J Neurosci.

[20] Tremblay R, Lee S, Rudy B (2016). GABAergic interneurons in the neocortex: from cellular properties to circuits. Neuron.

[21] Kantamneni S, Gonzalez-Gonzalez IM, Luo J, Clmarostl H, Jacobs SC, Jaafarl N, Henly JM (2014). Defferential regulation of GABA_B_ receptor trafficking by different modes of *N*-methyl-d-aspartate (NMDA) receptor signaling. J Biol Chem.

[22] Meldrum BS, Rogawski MA (2007). Molecular targets for antiepileptic drug development. Neurotherapeutics.

[23] Sun H, Wu SH (2009). The physiological role of pre- and postsynaptic GABA_B_ receptors in membrane excitability and synaptic transmission of neurons in the rat’s dorsal cortex of the inferior colliculus. Neuroscience.

[24] Thompson SM, Gähwiler BH (1992). Comparison of the actions of baclofen at pre- and postsynaptic receptors in the rat hippocampus *in vitro*. J Physiol.

[25] Booker SA, Harada H, Elgueta C, Bank J, Bartos M, Kulik A, Vida I (2020). Presynaptic GABA_B_ receptors functionally uncouple somatostatin interneurons from the active hippocampal network. Elife.

[26] Gassmann M, Bettler B (2012). Regulation of neuronal GABA_B_ receptor functions by subunit composition. Nat Rev Neurosci.

[27] Wu L-G, Saggau P (1995). GABA_B_ receptor-mediated presynaptic inhibition in guinea-pig hippocampus is caused by reduction of presynaptic Ca^2+^ influx. J Physiol.

[28] Deng PY, Xiao Z, Yang C, Rojanathammanee L, Grisanti L, Watt J, Geiger JD, Liu R, Porter JE, Lei S (2009). GABA_B_ receptor activation inhibits neuronal excitability and spatial learning in the entorhinal cortex by activating TREK-2 K^+^ channels. Neuron.

[29] Gähwiler BH, Brown DA (1985). GABAB-receptor-activated K^+^ current in voltage-clamped CA_3_ pyramidal cells in hippocampal cultures. Proc Natl Acad Sci USA.

[30] Chalifoux JR, Carter AG (2010). GABA_B_ receptors modulate NMDA receptor calcium signals in dendritic spines. Neuron.

[31] Chalifoux JR, Carter AG (2011). GABA_B_ receptors modulation of synaptic function. Curr Opin Neurobiol.

[32] Kaupmann K, Malitschek B, Schuler V, Heid J, Froestl W, Beck P, Mosbacher J, Bischoff S, Kulik A, Shigemoto R, Karschin A, Bettler B (1998). GABA_B_-receptor subtypes assemble into functional heteromeric complexes. Nature.

[33] Skeberdis VA, Chevaleyre V, Lau CG, Goldberg JH, Pettit DL, Suadicani SO, Lin Y, Bennett MVL, Yuste R, Castillo PE, Zukin RS (2006). Protein kinase A regulates calcium permeability of NMDA receptors. Nat Neurosci.

[34] Bliss TVP, Collingridge GL (1993). A synaptic model of memory: long-term potentiation in the hippocampus. Nature.

[35] Collingridge GL (1987). The role of NMDA receptors in learning and memory. Nature.

[36] Johnson LR, Battle AR, Martinac B (2019). Remembering mechanosensitivity of NMDA receptors. Front Cell Neurosci.

[37] Abbott LF, Nelson SB (2000). Synaptic plasticity: taming the beast. Nat Neurosci.

[38] Yoshimura H, Kato N, Sugai T, Segami N, Onoda N (2003). Age-dependent appearance of an insulo-parietal cortical signal propagation that elicits a synchronized population oscillation in the parietal cortex in rats. Dev Brain Res.

[39] Paxinos G, Watson C (1997). The rat brain in stereotaxic coordinates.

[40] Swanson LW (1992). Brain maps: structure of the rat brain.

[41] Zilles K, Wree A, Paxinos G (1995). Cortex. The rat nervous system.

[42] Yoshimura H, Kato N, Honjo M, Sugai T, Segami N, Onoda N (2004). Age-dependent emergence of parieto-insular corticocortical signal flow in developing rats. Brain Res.

[43] Yoshimura H, Kato N, Sugai T, Honjo M, Sato J, Segami N, Onoda N (2004). To-and-fro optical voltage signal propagation between the insular gustatory and parietal oral somatosensory areas in rat cortical slices. Brain Res.

[44] Gu N, Jackson J, Goutangny R, Lowe G, Manseau F, Williams S (2013). NMDA-development phase synchronization between septal and temporal CA3 hippocampal networks. J Neurosci.

[45] Cadonic C, Albensi A (2014). Oscillations and NMDA receptors: their interplay create memories. AIMS Neurosci.

[46] Tominaga Y, Taketoshi M, Maeda N, Tominaga T (2019). Wide-dield single-photon optical recording in brain slice using voltage-sensitive dye. J Vis Exp.

[47] Tominaga T, Tominaga Y, Yamada H, Matsumoto G, Ichikawa M (2000). Quantification of optical signals with electrophysiological signals in neural activities of Di-4-ANEPPS stained rat hippocampal slices. J Neurosci Methods.

[48] Tominaga Y, Taketoshi M, Tominaga T (2018). Overall assay of neuronal signal propagation pattern with long-term potentiation (LTP) in hippocampal slices from the CA1 area with fast voltage-sensitive dye imaging. Front Cell Neurosci.

[49] Gusain P, Taketoshi M, Tominaga T, Timinaga Y (2023). Functional dissection of ipsilateral and contralataral neural activity propagation using voltage-sensitive dye imaging in mouse prefrontal cortex. eNeuro.

[50] Yoshimura H, Sugai T, Onoda N, Segami N, Kato N (2001). Synchronized population oscillation of excitatory synaptic potentials dependent of calcium-induced calcium release in rat neocortex. Brain Res.

[51] Yoshimura H, Kato N, Sugai T, Segami N, Onoda N (2003). Age-dependent emergence of oscillatory signal flow between the primary and secondary visual cortices in rat brain slices. Brain Res.

[52] Yoshimura H, Mashiyama Y, Kaneyama K, Nagao T, Segami N (2007). Opening of shortcut circuits between visual and retro splenial granular cortices of rats. NeuroReport.

[53] Yoshimura H, Sugai T, Honjo M, Segami N, Onoda N (2005). NMDA receptor-dependent oscillatory signal outputs from the retrosplenial cortex triggered by s non-NMDA receptor-dependent signal input from the visual cortex. Brain Res.

[54] Yoshimura H, Sugai T, Hasegawa T, Yao C, Akamatsu T, Kato N (2013). Age-dependent emergence of caffeine-assisted voltage oscillations in the endopiriform nucleus of rats. Neurosci Res.

[55] Yoshimura H, Sugai T, Onoda N, Segami N, Kato N (2002). Age-dependent occurrence of synchronized population oscillation suggestive of a developing functional coupling between NMDA and ryanodine receptors in the neocortex. Dev Brain Res.

[56] Bartos M, Vida I, Frotscher M, Meyer A, Monyue H, Geiger JRP, Jonas P (2002). Fast synaptic inhibition promotes synchronized gamma oscillations in hippocampal interneuron networks. Proc Nat Acad Sci USA.

[57] Fredholm BB, Battig K, Holmen J, Nehlig A, Zuvartau EE (1999). Actions of caffeine in the brain with special reference to factors that contribute to its widespread use. Pharmacol Rev.

[58] Hosteler RE, Hu H, Agmon A (2023). Genetically defined subtypes of somatostatin-containing cortical interneurons. eNeuro.

[59] Xu H, Jeong H-Y, Tremblay R, Rudy B (2013). Neocortical somatostatin-expressing GABAergic interneurons disinhibit the thalamorecipient layer 4. Neuron.

[60] Kanigowski D, Bogaj K, Barth AL, Urban-Ciecko J (2023). Somatostatin-expressing interneurons modulate neocortical network through GABAb receptors in a synapse-specific manner. Sci Rep.

[61] Qian J, Noebels JL (2000). Presynaptic Ca2+ influx at a mouse central synapse with Ca^2+^ channel subunit mutations. J Neurosci.

[62] Wang CS, Monteggia LM, Kavalai ET (2023). Spatially non-overlapping Ca^2+^ signals drive distinct forms of neurotransmission. Cell Rep.

[63] Heine M, Heck J, Ciuraszkiewicz A, Bikbaev A (2020). Dynamic compartmentalization of calcium channel signaling in neurons. Neuropharmacology.

[64] Avila J, Lliorens-Martín M, Pallas-Bazarra N, Bolόs M, Perea JR, Rodriguez-Matellán A, Hernández F (2017). Cognitive decline in neuronal aging and Alzheimer’s disease: role of NMDA receptors and associated proteins. Front Neurosci.

[65] Fedele L, Newcombe J, Topf M, Gibb A, Haevey RJ, Smary TG (2018). Disease-associated missense mutations in GluN2B subunit alter NMDA receptor ligand binding and ion channel properties. Nat Commun.

[66] Zamzow DR, Elias V, Shumaker M, Larson C, Magnusson KR (2013). An increase in the association of GluN2B containing NMDA receptors with membrane scaffolding proteins was related to memory declines during aging. J Neurosci.

[67] Hardingham GE, Bading H (2010). Synaptic versus extrasynaptic NMDA receptor signaling: implications for neurodegenerative disorders. Nat Rev Neurosci.

[68] Bliss TVP, Collingridge GL (2013). Expression of NMDA receptor-dependent LTP in the hippocampus: bridging the divide. Mol Brain.

[69] Kato N, Yoshimura H (1993). Reduced Mg^2+^ block of *N*-methyl-d-aspartate receptor-mediated synaptic potentials in developing visual cortex. Proc Natl Acad Sci USA.

[70] Yoshimura H, Sugai T, Segami N, Onoda N (2005). Strengthening of non-NMDA receptor-dependent horizontal pathways between primary and lateral secondary visual cortices after NMDA receptor-dependent oscillatory neural activities. Brain Res.

[71] Lu W-Y, Man H-Y, Ju W, Trimble WS, MacDonald JF, Wang YT (2001). Activation of synaptic NMDA receptors induces membrane insertion of new AMPA receptors and LTP in cultured hippocampal neurons. Neuron.

[72] Qi G, van Aerde K, Abel T, Feldmeyer D (2017). Adenosine differentially modulates synaptic transmission of excitatory and inhibitory microcircuits in layer 4 of rat barrel cortex. Cereb Cortex.

[73] Yoshimura H (2005). The potential of caffeine for functional modification from cortical synapses to neuron networks in the brain. Curr Neuropharmacol.

[74] Evenseth LSM, Gabrielsen M, Sylte I (2020). The GABA_B_ receptor-structure, ligand binding and drug development. Molecules.

[75] Xu C, Zhang W, Rondard P, Pin J-P, Liu J (2014). Complex GABA_B_ receptor complexes: how to generate multiple functionally distinct units from a single receptor. Front Pharmacol.

[76] Carletti R, Tacconi S, Mugnaini M, Gerrard P (2017). Receptor distribution studies. Curr Opin Pharmacol.

[77] Fritschy J-M, Meskenaite V, Weinmann O, Honer M, Benke D, Mohler H (1999). GABA_B_-receptor splice variants GB1a and GB1b in rat brain: developmental regulation, cellular distribution and extrasynaptic localization. Eur J Neurosci.

[78] Kulik Á, Vida I, Lujan R, Haas CA, Lopez-Bendito G, Shigemoto R, Frotscher M (2003). Subcellular localization of metabotropic GABA_B_ receptor subunit GABA_B1a/b_ and GABA_B2_ in the rat hippocampus. J Neurosci.

[79] Lόpez-Bendito G, Shigemoto R, Kulik A, Vida I, Fairen A, Lujan R (2004). Distribution of metabotoropic GABA receptor subunit GABA_B1a/b_ and GABA_B2_ in the rat hippocampus during prenatal and postnatal development. Hippocampus.

[80] Vogt R, Barbieri S, Brauner-Osborne H, Turecek R, Shigemoto R, Zhang Y-P, Luján R, Jacobson LH, Biemann B, Fritschy J-M, Vacher C-M, Müller M, Sansig G, Guetg N, Cryan JF, Kaupmann K, Gassmann M, Oertner TG, Bettler B (2006). Differential compartmentalization and distinct functions of GABA_B_ receptor variants. Neuron.

[81] Yoshimura H, Honjo M, Mashiyama Y, Kaneyama K, Segami N, Sato J, Sugai T, Kato N, Onoda N (2008). Multiple tooth-losses during development suppress age-dependent emergence of oscillatory neural activities in the oral somatosensory cortex. Brain Res.

[82] Fukuda T, Tominaga T, Tominaga Y, Kanayama H, Kato N (2023). Alternative strategy for driving voltage-oscillator in rat neocortex of rats. Neurosci Res.

[83] Bullier J (2001). Integrated model of visual processing. Brain Res.

[84] Alexander AS (2023). Rethinking retrosplenial cortex: Perspectives and predictions. Neuron.

